# Exploring the knowledge, attitude, and practice of community pharmacists regarding pediatric asthma management in Guangdong Province, China: a cross-sectional survey study

**DOI:** 10.1186/s12909-025-06885-6

**Published:** 2025-02-22

**Authors:** Guohua Lin, Meijun Lai, Chi Ian Chau, Hao Hu, Carolina Oi Lam Ung

**Affiliations:** 1 Institute of Chinese Medical Sciences, University of Macau, Macao SAR, China; 2 Guangyao Pharmacy (Guangdong) Co, Ltd, Guangzhou, China; 3 Centre for Pharmaceutical Regulatory Sciences, University of Macau, Macao SAR, China; 4 Department of Public Health and Medicinal Administration, Faculty of Health Sciences, University of Macau, Macao SAR, China

**Keywords:** Asthma, Community pharmacist, Pediatrics, Survey

## Abstract

**Background:**

Pediatric asthma is a common respiratory disease that significantly affects children's physical and mental health. This study aims to investigate community pharmacists' self-reported knowledge of asthma and explore their attitude and practice in providing pharmaceutical services to address the needs of pediatric asthma patients.

**Methods:**

An online questionnaire informed by recent literature was distributed to community pharmacists in Guangdong Province, China via Qualtrics using convenience sampling and snowballing. Descriptive analysis and generalized multiple linear regression analysis were used for data analysis.

**Results:**

Out of 579 community pharmacists who attempted this questionnaire, 473 completed it, giving a completion rate of 81.7%. Among the participants, 217 (45.9%) were female, and 319 (67.5%) aged between 31 and 50. The mean score of self-reported knowledge was 60.29 ± 6.16 out of 95 (range: 19–76); the mean score of attitude was 87.62 ± 8.37 out of 130 (range: 77–130); the mean score of practice was 18.72 ± 2.32 out of 30 (range: 6–24). Age, years of employment, highest education level, the average number of patients serving per day, and the average number of asthma patients serving per week were factors significantly associated with participants' self-reported knowledge, attitude, or practice toward pediatric asthma management (*p* < 0.05).

**Conclusions:**

Many community pharmacists felt they lacked sufficient knowledge about pediatric asthma and infrequently provided pharmaceutical services to children with asthma, despite having a positive attitude towards this professional role. To enhance the pharmaceutical care for pediatric asthma patients, it is essential to develop practice guidelines and care models, and provide education and training to community pharmacists accordingly.

## Background

Pediatric asthma is a common respiratory disease that significantly impacts the physical and mental health of children [1]. Approximately 14% of children worldwide are affected by asthma, with its prevalence on the rise. Pediatric asthma may also lead to mortality especially in low-income and middle-income countries, imposing substantial burden on both individuals and healthcare systems [2]. Asthma is one of the top 10 causes of disability-adjusted life years in children of aged 5–14 [3] and is one of the primary reasons for school absenteeism [4]. Moreover, it is one of the main causes of hospitalization in children, occurring at least twice as frequently as in adults, which contributes significantly to global asthma-related costs [5].

Pediatric asthma is a heterogeneous disease with phenotypes that may vary over time. To achieve adequate management, continuous observation of symptoms and timely adjustment of treatment are necessary [6]. However, many caregivers of pediatric asthma patients have limited knowledge about asthma, which often results in poor asthma control [7]. Studies have shown that educating caregivers of pediatric asthma patients on asthma-related knowledge or providing them with written asthma action plans can improve outcomes for pediatric asthma patients [8, 9].

As key players in primary healthcare, community pharmacists are more readily accessible to the patients compared with many other healthcare practitioner [10]. A meta-analysis of 771 intervention trials showed that the most effective interventions to improve patients’ medication adherence in chronic diseases like asthma were face-to-face, direct-to-patient services provided by pharmacists [11, 12]. Existing studies have shown that interventions led by community pharmacists can improve various outcomes for pediatric asthma patients, including increased asthma control rates, improved lung function, and better inhaler technique [13–15]. However, research evaluating their knowledge, attitude, and practice regarding pediatric asthma management remains limited [16, 17].

This study aims to investigate community pharmacists' self-reported knowledge of asthma and explore their attitudes and practices in providing pharmaceutical services for pediatric asthma care in Guangdong Province, China. Given the high prevalence and significant burden of pediatric asthma in the area, the findings of this study are expected to contribute to enhanced pediatric asthma management locally and in similar regions.

## Methods

### Study design and participants

This was a cross-sectional survey-based study. Community pharmacists in Guangdong Province, China were eligible for inclusion in this study. According to the National Bureau of Statistics, the number of pharmacists in Guangdong Province, China in 2023 was 52,400 [18]. Therefore, the minimum sample size required was determined to be 382 (confidence level 95%, margin of error 5%). The data was collected from April to May 2024 using convenience sampling and snowballing. A poster containing a brief introduction and a link to the online survey was used to invite participants. The Guangdong Pharmaceutical Association facilitated the distribution of the poster by sharing it to its member pharmacies via social media platforms and professional chat groups. The online questionnaire was hosted by Qualtrics. All information was collected anonymously.

### Questionnaire design

The questionnaire design was informed by a previous literature review summarizing pharmacist interventions for pediatric asthma [19]. At the start of the questionnaire, participants were required to confirm their status as pharmacists whose primary workplace was a community pharmacy before proceeding to the subsequent questions. The questionnaire consisted of 4 sections, including participant demographics (9 questions), self-reported knowledge related to pediatric asthma care (19 statements), attitude towards the provision of pharmaceutical services for pediatric asthma (26 statements), and practice of pharmaceutical services related to pediatric asthma (6 statements). Each page of the questionnaire included a "Previous" button, enabling participants to view and modify their answers at any time. A reminder function was employed to ensure participants completed all questions on the page before proceeding to the next. The estimated completion time for the questionnaire was 15 to 20 min.

Participants were asked to respond using a 5-point Likert scale to the statements in the knowledge Sect. (1 = very insufficient; 2 = insufficient; 3 = unclear; 4 = sufficient; 5 = very sufficient), the attitude Sect. (1 = very disagree; 2 = disagree; 3 = unclear; 4 = agree; 5 = very agree), and the practice Sect. (1 = never, 2 = Rarely, 3 = Sometimes, 4 = often, 5 = always). It is worth noting that the statements in the attitude section were designed to correspond to 6 aspects of services, which align with the 6 practice areas in the practice section: disease education, medication supply, asthma management guidance, medication use guidance, non-pharmacological intervention guidance, and follow-up care. The total score for each section was the sum of all question scores. Consequently, the score minimum and maximum ranges for the knowledge, attitude, and practice sections were 19–95, 26–130, and 6–30, respectively. Higher scores would indicate better self-reported knowledge, more positive attitude, and more frequent practice.

In order to ensure the readability of the questionnaire content, a pilot study was conducted, in which 21 community pharmacists participated. Following the pilot, all the questions in the questionnaire were retained, but the wording of some questions was modified to improve clarity. The Cronbach’s alpha values for those subscales were acceptable (knowledge: Cronbach's α = 0.958; attitudes: Cronbach's α = 0.955; practices: Cronbach's α = 0.874), indicating good internal consistency. Additionally, the Kaiser–Meyer–Olkin value of 0.605 indicated a highly acceptable score with a significant Bartlett's test of sphericity (*p* < 0.001).

### Statistical analysis

Excel and IBM SPSS version 24.0 were used for data analysis. Descriptive statistics were employed to describe the demographic characteristics of the participants, as well as their self-reported knowledge related to pediatric asthma, attitude towards pharmaceutical services for pediatric asthma, and practices in providing pharmaceutical services for pediatric asthma. Continuous variables were expressed as means and standard deviations, while categorical data were presented as frequencies and percentages.

The Kolmogorov–Smirnov test was performed to test data normality. For continuous variables with non-normal distributions, univariate analyses were conducted using the Mann–Whitney U test or Kruskal–Wallis test, as appropriate. To identify factors associated with community pharmacists' knowledge, attitudes, and practices regarding pediatric asthma management, three generalized linear regression models were developed. Statistical significance was set at *p*-values < 0.05.

### Ethical approval

The study was reviewed and approved by the University of Macau research committee (Ethics Assessment ID: SSHRE24-APP044-ICMS). Consent was obtained from the participants at the beginning of the questionnaire.

## Results

### Participants’ demographic characteristics

A total of 579 community pharmacists accessed this online survey, of whom 473 agreed and completed the questionnaire, giving a completion rate of 81.7%. The demographic characteristics of the participants are shown in Table 1. Among the participants, 45.9% were women. The majority of the participants aged between 31 and 50 years, accounting for 67.5%. There were 22.0%, 23.0% and 48.8% of participants who had worked for 4–10 years, 11–20 years, and more than 20 years, respectively. The highest level of education of most participants was bachelor's degree, accounting for 74.0%. The majority of participants served 10–50 patients per day, accounting for 66.6%, followed by 51–100, accounting for 23.6%. In terms of the number of asthma patients served per week, 57.5% and 22.0% of participants reported serving 51–100 and over 100 asthma patients per week, respectively. Only a small number of participants had a family history of asthma, accounting for 6.8%.

**Table 1:** Demographics characteristics of participants (*n* = 473)

Demographic characteristics	Cases	%
Gender		
Female	217	45.9%
Male	256	54.1%
Age (years)		
≤ 30	68	14.3%
31–40	165	34.9%
41–50	154	32.6%
> 50	86	18.2%
Years of employment		
≤ 3	29	6.1%
4–10	104	22.0%
11–20	109	23.0%
> 20	231	48.8%
Highest education level		
Bachelor	350	74.0%
Master or PhD	100	21.1%
Others	23	4.9%
The average number of patients serving per day		
< 10	46	9.7%
10–50	315	66.6%
> 50	112	23.6%
The average number of asthma patients serving per week		
< 10	56	11.8%
10–50	41	8.7%
51–100	272	57.5%
> 100	104	22.0%
Any history of asthma of you/your family		
Yes	32	6.8%
No	441	93.2%

### Participants’ self-reported knowledge in pediatric asthma

**Fig. 1:**
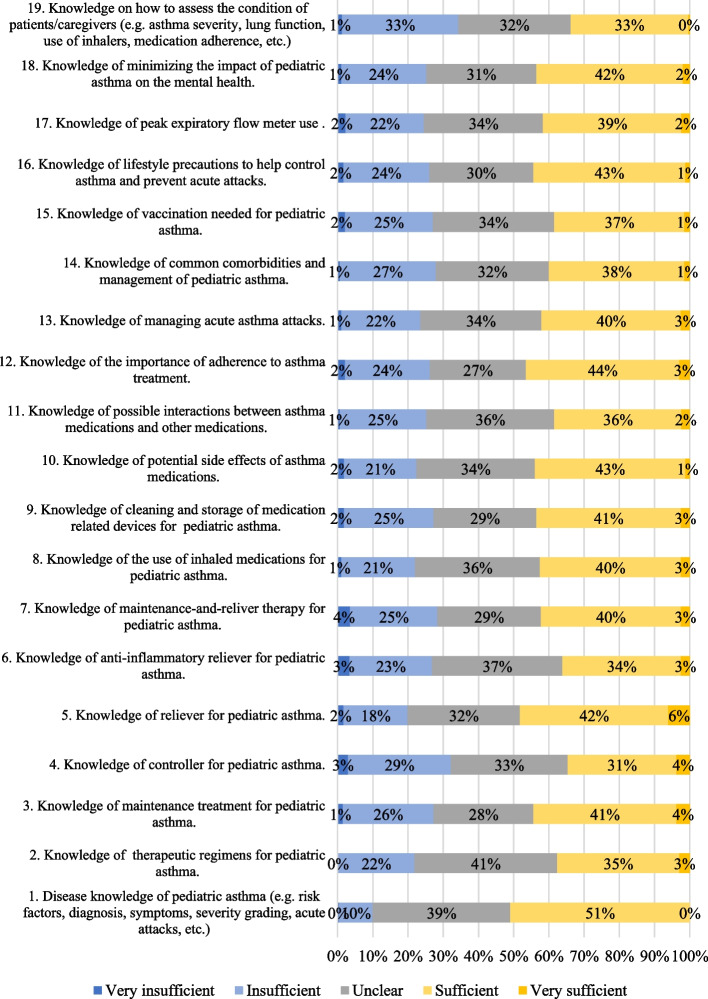
Participants’ self-reported knowledge in pediatric asthma

The details of self-reported knowledge in pediatric asthma perceived by the participants are shown in Fig. 1. Only 33–51% of the participants agreed or strongly agreed that they had sufficient knowledge in various aspect of pediatric asthma. Up to 34% believed otherwise. Areas of knowledge which participants mostly considered sufficient or very sufficient included “*Item 1—Disease knowledge of pediatric asthma*” (51%), “*Item 5—Reliever medication for asthma*” (48%), and “*Item 12—Importance of adherence to asthma treatment*” (47%). It is also worth noting that the areas of knowledge which pharmacists mostly found lacking was “*Item 19—How to assess the conditions of the patients*” (34%) and “*Item 4—Controller medication for asthma*” (32%). Between 27–41% were not certain if they had sufficient knowledge in various aspects of pediatric asthma.

### Participants’ attitudes towards providing pharmaceutical services for pediatric asthma

**Fig. 2:**
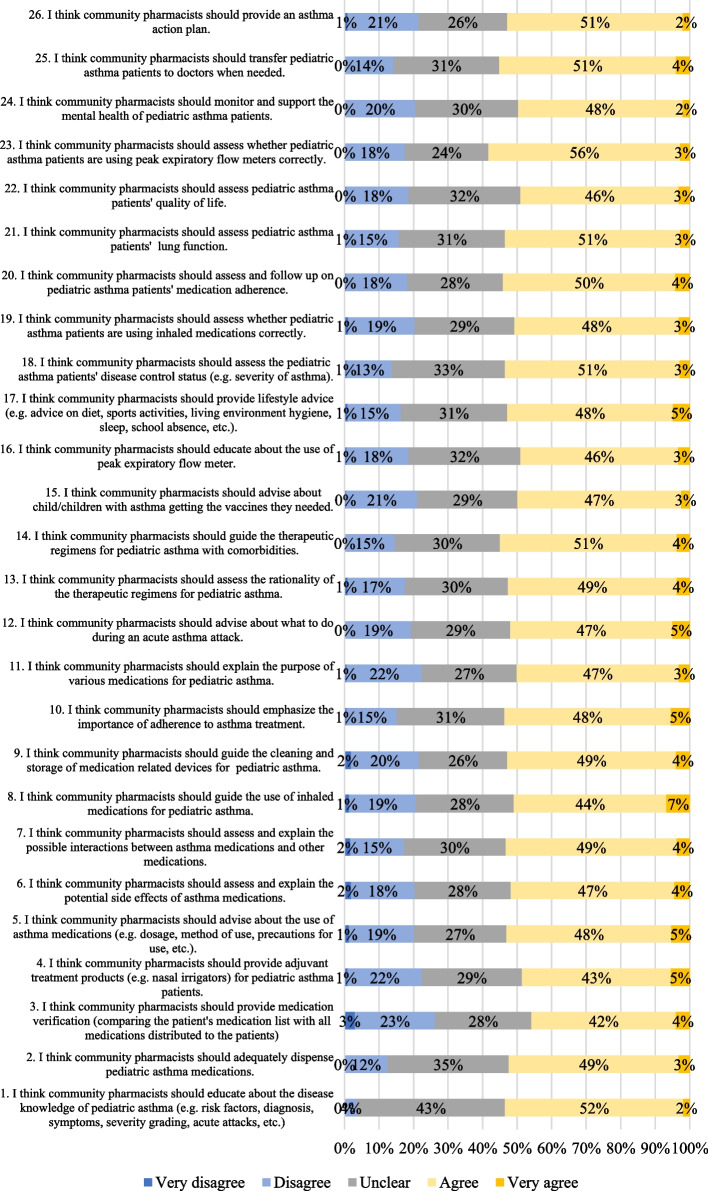
Participants’ attitude towards providing pharmaceutical services in pediatric asthma

The scores of the participants’ attitudes towards providing pharmaceutical services for pediatric asthma are shown in Fig. 2. Around 49–59% of the participants agreed or strongly agreed that they should provide professional services related to the care for pediatric asthma patients. Areas of pharmaceutical services which most participants agreed or strongly agreed on were “*Item 23—Assess whether pediatric asthma patients are using peak expiratory flow meters correctly*” (59%), “*Item 14—Guide the therapeutic regimens for pediatric asthma with comorbidities*” (55%), and “*Item 1—Educate about the disease knowledge of pediatric asthma*” (54%). It is also worth noting that the type of pharmaceutical services which pharmacists mostly disagreed on were “*Item 3—Provide medication verification*” (26%), “*Item 4—provide adjuvant treatment products for pediatric asthma patients*” (23%), and “*Item 11—explain the purpose of various medications for pediatric asthma*” (23%). Between 26–43% of the participants were not certain if they should provide pharmaceutical services to pediatric asthma patients.

*As explained earlier, the statements in the attitude section aligned with the 6 aspects of pharmaceutical care in pediatric care identified in the literature which included: disease education (Item 1), medication supply (Items 2–4), medication use guidance (Items 5–10), asthma management guidance (Items 11–14), non-pharmacological intervention guidance (Item 15–17), and follow-up care (Items 18–26).* In terms of disease education, 54% the participants believed that they should educate about the disease of pediatric asthma, while 4% thought that they should not. In terms of medication supply, 52% of the participants thought that they should adequately dispense pediatric asthma medications, while relatively fewer participants thought that they should provide medical verification and provide advanced treatment products, accounting for 46% and 48% the participants respectively.

In terms of providing guidance on medication use, more consistent results were collected. Over 50% participants consistently agreed that they should advise the patients how to use the asthma medication, assess and explain potential sides effects of the asthma medication and possible drug interactions, guide the use, learning and storage of inhaler medication, and educate patient the importance of medication adherence. In terms of providing guidance on the overall pediatric asthma management, services which participants mostly agreed on were “*Item 14—Guide the therapeutic regimens for pediatric asthma with comorbidities*”, accounting for 55%. However, explaining the purpose of various medicine for pediatrics asthma (*Item 11*) was mostly disagreed on by the participants (23%).

In terms of non-pharmacological interventions, 53% of the participants were positive that they should provide lifestyle advice (*Item 17*). Relatively fewer participants thought that they should provide education on the use of peak expiratory flow meter (*Item 16*) and advise about child/children with asthma getting the vaccines they needed (*Item 15*), accounting for 49% and 50% respectively. In terms of follow-up patient care, most participants (59%) thought that they should assess whether pediatric asthma patients were using peak expiratory flow meters correctly (*Item 19*). Compared with other interventions of patient follow-up, only 49% participants agreed that they should assess pediatric asthma patients' quality of life was lower (*Item 22*).

### Participants’ practice related to pediatric asthma

**Fig. 3:**
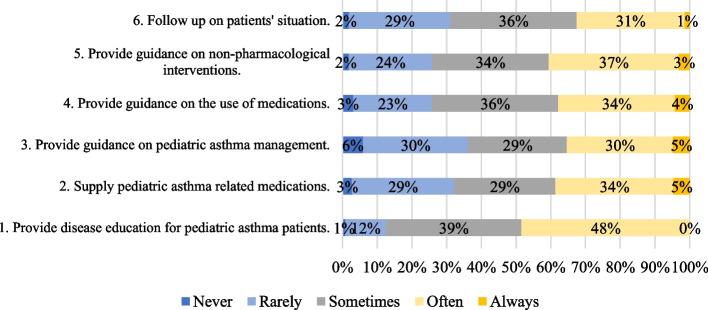
Participants’ practice related to pediatric asthma

The results about the participants’ practice in relation to pediatric asthma patients are shown in Fig. 3. Only 32–48% of the participants reported that they often or always provide various professional services related to the care for pediatric asthma patients. Areas of services which most participants indicated they frequently provided included “*Item 1 – Provide disease education*” (48%), “*Item 5—Provide guidance on non-pharmacological interventions*” (40%), and “*Item 2—Supply asthma medications*” (39%). It is also worth noting that the type of pharmaceutical services which participants rarely or even never provided were “*Item 3—Provide guidance on pediatric asthma management*” (36%). Between 29–39% indicated they only provided pharmaceutical services to pediatric asthma patients occasionally.

### Factors associated with participants’ self-reported knowledge, attitude, and practice

As shown in Table 2, the mean score of self-reported knowledge was 60.29 ± 6.16 out of 95 (range: 19–76); the mean score of attitude was 87.62 ± 8.37 out of 130 (range: 77–130); and the mean score of practice was 18.72 ± 2.32 out of 30 (range: 6–24).

**Table 2:** Participants’ scores of self-reported knowledge, attitude, and practice (*n* = 473)

Section	Number of items	Full scores	Participants’ score		
			**Mean ± SD**	**Lowest scores**	**Highest scores**
Knowledge	19	95	60.29 ± 6.16	19	76
Attitudes	26	130	87.62 ± 8.37	77	130
Practices	6	30	18.72 ± 2.32	6	24

### Factors associated with participants’ self-reported knowledge, attitude and practice

Three generalized multiple linear models were established to explore the factors associated with participants’ self-reported knowledge, attitude and practice regarding pediatric asthma management, respectively. As shown in Table 3, among all the demographic characteristics tested, variables related to the participants’ experiences were associated with statistically differences in their self-reported knowledge, attitude and practice. It was shown that the independent variables: age (*p* < 0.05), employment less than 3 years (B = −4.402, *p* = 0.032), highest education level (*p* < 0.05), the average number of asthma patients serving per day less than 10 patients (B = −4.683, *p* = 0.015), and the average number of asthma patients serving per week less than 10 patients (B = −8.990, *p* < 0.001) demonstrated significant associations with the participants’ self-reported knowledge. The independent variables: employment less than 3 years (B = −6.116, *p* = 0.001), highest education level of Bachelor (B = −1.594, *p* = 0.005) the average number of patients serving per day less than 10 patients (*p* < 0.05), and the average number of asthma patients serving per week (*p* < 0.05) demonstrated significant associations with the participants’ attitude. The independent variables: the average number of patients serving per day less than 10 patients (B = −2.167, *p* = 0.003), and the average number of asthma patients serving per week (*p* < 0.05) demonstrated significant associations with the participants’ frequency of practice.

**Table 3:** Generalized multiple linear regression analysis between various factors and the total score of participants’ self-reported knowledge, attitude and practice

Variables	Knowledge		Attitude		Practice	
	**B**	***p***	**B**	***p***	**B**	***p***
Gender						
Female	−0.176	0.730	0.169	0.719	−0.315	0.096
Male	Ref		Ref		Ref	
Age (years)						
≤ 30	4.562	0.017*	2.602	0.140	−0.302	0.673
31–40	4.562	0.010*	0.330	0.828	−0.323	0.600
41–50	0.900	0.203	−0.622	0.337	0.231	0.379
> 50	Ref		Ref		Ref	
Years of employment						
≤ 3	−4.402	0.032*	−6.116	0.001*	0.138	0.857
4–10	−3.083	0.068	−1.859	0.231	0.658	0.295
11–20	−2.984	0.054	−0.368	0.796	0.687	0.234
> 20	Ref		Ref		Ref	
Highest education level						
Others	−4.122	0.004*	−0.975	0.457	−0.443	0.404
Bachelor	−1.749	0.005*	−1.594	0.005*	−0.154	0.507
Master or PhD	Ref					
The average number of patients serving per day						
< 10	−4.683	0.015*	−11.156	0.000*	−2.167	0.003*
10–50	−1.083	0.534	−3.417	0.033*	−0.685	0.291
> 50	Ref		Ref		Ref	
The average number of asthma patients serving per week						
< 10	−8.990	0.000*	−26.318	0.000*	−4.638	0.000*
10–50	−2.689	0.177	−17.187	0.000*	−2.040	0.006*
51–100	−0.379	0.835	−4.194	0.012*	−0.460	0.497
> 100	Ref		Ref		Ref	
Any history of asthma of you/your family						
Yes	1.317	0.177	0.569	0.525	0.057	0.875
No	Ref		Ref		Ref	

^*^p < 0.05

## Discussion

This study revealed that many community pharmacists believed they did not fully master the pediatric asthma knowledge (mean score: 60.29 out of 95, score range: 19–95). However, many of them held a positive attitude towards providing pharmaceutical services for pediatric asthma (mean score: 87.62 out of 130, score range: 26–130). Notably, the frequency of them providing such services in practice was relatively low (mean score: 18.72 out of 30, score range: 6–30). The results of the three generalized multiple linear models revealed that age, experiences of practice (years of employment), highest education level, and opportunities to provide direct-to-patient care (including the average number of patients serving per day and the average number of asthma patients serving per week) were significantly associated with their self-reported knowledge, attitudes, and practices regarding pediatric asthma management (*p* < *0.05*). These findings underscore the need to enhance education and training for community pharmacists in pediatric asthma management and to provide an enabling environment for them to practice and improve their pharmaceutical services.

The findings about community pharmacists not having sufficient pediatric asthma knowledge are in agreement with previous studies. A study in Jordan found that 42.9% of the community pharmacists had low level of knowledge about asthma management [17]. Another study in France which investigated the knowledge of asthma management and inhalation techniques among community pharmacists found that, while community pharmacists’ technique of using inhaler medication had improved over time, their knowledge of asthma management remained at an average level [20]. Similarly, a cross-sectional survey in Nigeria that investigated community pharmacists' understanding of the Global Initiative for Asthma (GINA) report showed that only 34.8% of community pharmacists had a good understanding of asthma [21].

The results about community pharmacists' attitudes towards providing asthma management in this study also aligned with previous findings. In this study, community pharmacists held a positive attitude towards providing pharmaceutical services for pediatric asthma. Another study in Egypt presented similar results and found that 100% of pharmacists exhibited a positive attitude towards asthma management [22]. In addition, another study in Jordan which investigated community pharmacists’ attitudes towards asthma management and counseling showed that more than half of the participants (55.5%) were in the positive attitude group [17]. These findings suggest that community pharmacists are subjectively willing to provide pharmaceutical services for pediatric asthma patients.

The findings regarding pharmacists' practices in pediatric asthma management in this study are somewhat inconsistent with previous studies. This study found that community pharmacists did not frequently provide pharmaceutical services for children with asthma. Similarly, the results of a study in Egypt revealed that only 13.6% pharmacists frequently provided pharmaceutical services for children with asthma, and the practice score of community pharmacists was significantly lower than that of hospital pharmacists [23]. However, the study in Jordan showed that 54.8% of community pharmacists demonstrated high frequency practice in pediatric asthma management [17]. Nevertheless, these findings consistently suggested the need to further explore and develop the role of community pharmacists in pediatric asthma management and allow them to practice their skills in their daily practice.

It is worth noting that both this study and the study in Jordan indicated that community pharmacists were least engaged in the follow-up management of pediatric asthma patients [17]. Pediatric asthma, as a chronic disease, is characterized by heterogeneity. It is necessary to conduct long-term monitoring and review of symptoms and treatment [23]. An observational study in the United States of America showed that 14-day follow-up after asthma emergency department visits was associated with a decrease in the rate of emergency department revisits [24]. As key players of the primary healthcare providers who are highly accessible to the patients, community pharmacists are well-positioned to significantly contribute to asthma control and the prevention of exacerbation through regular monitoring of the condition, medication review, and patient education during follow-up care [10].

In order to optimize asthma-related pharmaceutical services provided by community pharmacists, a number of actions may be considered. A study in Vietnam developed and implemented a training program for community pharmacists, and evaluated asthma knowledge and inhaler technique after training. The results showed that the training program significantly improved community pharmacists' asthma knowledge score, from 5.3 points to 17.2 points [25]. Turkey developed a project of asthma/COPD module, which was implemented nationwide at community pharmacies. Following the implementation of the project, there was a significant improvement in the patients’ outcome parameters related to asthma, including peak flow rate, asthma control test, and inhaler technique [26].

Furthermore, previous studies showed that family caregivers also needed the support as many of them were found to have insufficient knowledge about asthma, which was later found associated with poor asthma control in children [27–29]. The number of doctors are often limited especially in China [28]. A recent study showed that the number of pediatricians per child in the United States was almost twice that of China [30]. Given these challenges, fully leveraging the role of community pharmacists has the potential to improve the outcomes of pediatric asthma patients, and alleviating the doctors’ workload at the same time. Community pharmacists are expected to support pediatric asthma management by guiding patients on the proper use of inhalers, emphasizing the importance of medication adherence, and advising on the symptoms of asthma exacerbation [13–15].

This study also has some limitations. This study only collected questionnaires in the Guangdong Province. Future studies should draw nationally representative population sample for the study results to be adequately generalized to a larger context. In addition, community pharmacists who agreed to participate might be more interested in asthma management, which might have affected the representativeness of the study sample. Moreover, the results of community pharmacists’ knowledge were based on the self-reported responses to the questionnaires in this study and might be subject to recall bias. Future research can be designed to include observational studies to investigate community pharmacists’ performance during practice.

## Conclusion

Pediatric asthma management remains a challenge for many countries, including China. As key players of healthcare providers in the community, the role of community pharmacists in managing pediatric asthma should not be overlooked. At present, community pharmacists did not believe that they had sufficient knowledge about pediatric asthma, and they infrequently provided pharmaceutical services to children with asthma in China, despite their positive attitude. It is necessary to develop a practice guideline or care model and implement training for community pharmacists regarding pediatric asthma management. Enhancing education and training for community pharmacists in this area and allowing them the opportunities to practice will better equip them to provide pharmaceutical services for pediatric asthma patients more effectively in the future.

## Data Availability

The data analyzed in this study are available by request from the first author.
